# Association between sarcopenia and hemoglobin level: a systematic review and meta-analysis

**DOI:** 10.3389/fmed.2024.1424227

**Published:** 2024-07-25

**Authors:** Hui Wang, Ping Lin

**Affiliations:** Department of Geriatrics, Hangzhou Third People’s Hospital, Hangzhou, China

**Keywords:** anemia, hemoglobin level, sarcopenia, aging, muscle mass

## Abstract

**Background:**

Sarcopenia is a disease characterized by decreased skeletal muscle mass and function in elderly individuals. Decreased hemoglobin levels is a marker of anemia. According to reports, there may be an association between anemia and sarcopenia, but research is inconsistent. Therefore, this meta-analysis aims to explore the association between sarcopenia and low hemoglobin levels.

**Methods:**

We searched PubMed, Embase, the Cochrane Library, Web of Science, Ovid, China National Knowledge Infrastructure (CNKI), and Wan Fang databases until September 2022. The present study included cross-sectional and case-control studies regarding low hemoglobin levels and sarcopenia. The studies were selected using inclusion and exclusion criteria. Studies were meta-analyzed by Review Manager 5.4 and Stata 16.0. We performed the heterogeneity test using the *I*^2^ test. Subgroup analysis was carried out to explore the cause of heterogeneity. Egger test was used to evaluate publication bias.

**Results:**

Out of 1,550 initial studies, 16 studies were meta-analyzed. Sarcopenia participants had significantly lower levels of hemoglobin than controls (MD = −0.53, 95% CI: −0.68 to −0.37, *p* < 0.001). Subgroup analysis, performed in China population reported lower hemoglobin levels in the sarcopenia population (MD = −0.49, 95% CI: −0.65 to −0.33, *p* < 0.001). And sarcopenia based on AWGS criteria reported lower hemoglobin levels (MD = −0.49, 95% CI: −0.65 to −0.33, *p* < 0.001). Among the population from hospitals and communities, patients with sarcopenia have lower hemoglobin levels.

**Conclusion:**

Our meta-analysis found evidence that sarcopenia is associated with low hemoglobin levels. However, further large-scale prospective studies should be conducted in the future to further confirm our conclusions.

**Systematic review registration:**

PROSPERO, CDR42024532252.

## 1 Introduction

Sarcopenia, defined as the age-related reduction in lean muscle mass and muscle function (1), is linked to falls, fractures, disabilities, diminished quality of life, and imposes a greater economic burden along with increased healthcare expenses (2). Epidemiological surveys indicate that the global prevalence of sarcopenia varies between 10 and 27% (3), with the prevalence rate among elderly people in Asia ranging from 2.5 to 45.7% (4). With the development of an aging society, the prevalence of sarcopenia is increasing. Due to the serious harm and high incidence of sarcopenia, it has brought a heavy burden to an aging society.

Sarcopenia’s risk factors are diverse, including aging, illness, malnutrition, and sedentary lifestyle, among others (5). The nutritional status of the elderly declines over time, leading to reduced physical activity and a subsequent decline in muscle mass and function, consequently heightening the risk of sarcopenia. Likewise, persistent chronic inflammation, characterized by muscles experiencing oxidative stress, can further worsen the weakening of muscle strength (6).

Anemia presents a well-established hazard for frailty, reduced quality of life, and increased mortality among older individuals (7). The occurrence of anemia in the elderly can result from various factors including iron deficiency, chronic inflammation, or chronic kidney disease (8). Research has shown that anemia is associated with diminished muscle strength, physical dysfunction, reduced mobility, heightened disability risk, and mortality increase (9, 10). Decreased hemoglobin levels may impair the transportation of oxygen to skeletal muscles, thus compromising muscle strength (11). Chronic inflammation frequently leads to anemia, potentially impacting muscle mass and physical function negatively (6). Given the shared underlying mechanisms of sarcopenia and anemia, a correlation between the two is plausible.

However, there has been controversy over the relationship between sarcopenia and anemia in current research. Tseng’s et al. (12) study had analysis 730 patients that there was a significant association between anemia and sarcopenia; but Kitamura et al. (13) and Ko’s et al. (14) studies have shown that there is no discernible correlation between anemia and sarcopenia. These studies showed a controversial relationship between hemoglobin and sarcopenia, which prompted us to explore the relationship. Therefore, we investigated the association between low hemoglobin level and sarcopenia by conducting a thorough meta-analysis, with diverse subgroup analyses to elucidate. The results will provide enhanced insights for the prevention and management of sarcopenia, thereby contributing to the amelioration of elderly health conditions.

## 2 Methods

This research was conducted according to the Preferred Reporting Items for Meta-Analysis (PRISMA) guidelines. It is registered in the International Prospective Register of Systematic Reviews (PROSPERO) with the registration number CDR42024532252.

### 2.1 Literature search

Two researchers independently searched PubMed, Embase, the Cochrane Library, Web of Science, Ovid, China National Knowledge Infrastructure (CNKI), and Wan Fang to identify relevant papers published before March 2024. The search terms included sarcopenia, anemia, hemoglobin, hyphemia, and low-level hemoglobin. To ensure comprehensive coverage, we carefully reviewed all eligible studies for inclusion and examined referenced reviews to avoid any potentially missed papers. In cases where multiple publications existed for the same clinical trial, we included only the most informative or up-to-date publication.

### 2.2 Selection criteria

In this systematic review, we employed the following inclusion criteria: (1) diagnosis of sarcopenia based on European Working Group on Sarcopenia in Older People (EWGSOP), Asian Working Group for Sarcopenia (AWGS), consensuses, or any other definition provided by original studies’ author; (2) the variables of the studies including serum or plasmatic levels of hemoglobin in both sarcopenia and control group (non-sarcopenic population) reported. Exclusion criteria comprised: (1) unclear reporting of sarcopenia diagnosis; (2) literature reviews, case reports, animal studies, or conference abstracts; (3) absence of quantitative hemoglobin level data.

### 2.3 Data extraction and confirmation

Two researchers independently extracted the following variables from the included studies: first author, publication year, location or nationality, study design, total number of individuals included in the study, gender distribution, applied definition of sarcopenia, method used for sarcopenia identification, mean muscle mass, handgrip strength, and gait speed in sarcopenia and group, hemoglobin levels in sarcopenia and non-sarcopenia population. Data verification was conducted by two researchers to reduce errors, with any discrepancies resolved through discussion or consultation with external sources.

### 2.4 Literature quality evaluation

We assessed the methodological quality of the included studies using the Newcastle−Ottawa scale (NOS) (15) for case-control studies and a modified version of the NOS for cross-sectional studies. This scale assessed studies based on three key dimensions: selection of study population, comparability of groups, and description of the outcome. The scale scores varied depending on the study design. For case-control studies, it ranged from 0 to 9 points with ≥7 points classified as high quality. For cross-sectional studies, it ranged from 0 to 7 points with ≥4 points considered as high quality. When any disagreement arose during data extraction and quality assessment, the two reviewers reached a consensus through negotiation.

### 2.5 Statistical analysis

All statistical analyses were performed by Review Manager 5.4 and Stata 16.0. The heterogeneity of the studies was analyzed using the *I*^2^ test. When *I*^2^ was higher than 50%, the random effect model was used; when *I*^2^ was less than 50%, the fixed effect model was performed. Subgroup analysis was carried out to explore the cause of heterogeneity. In addition, funnel plots were used initially to evaluate visual publication bias while Egger’s regression test was used to inferentially evaluate publication bias. *p*-values <0.05 was considered statistically significant (two-sided).

## 3 Results

### 3.1 Search results

**Figure 1:**
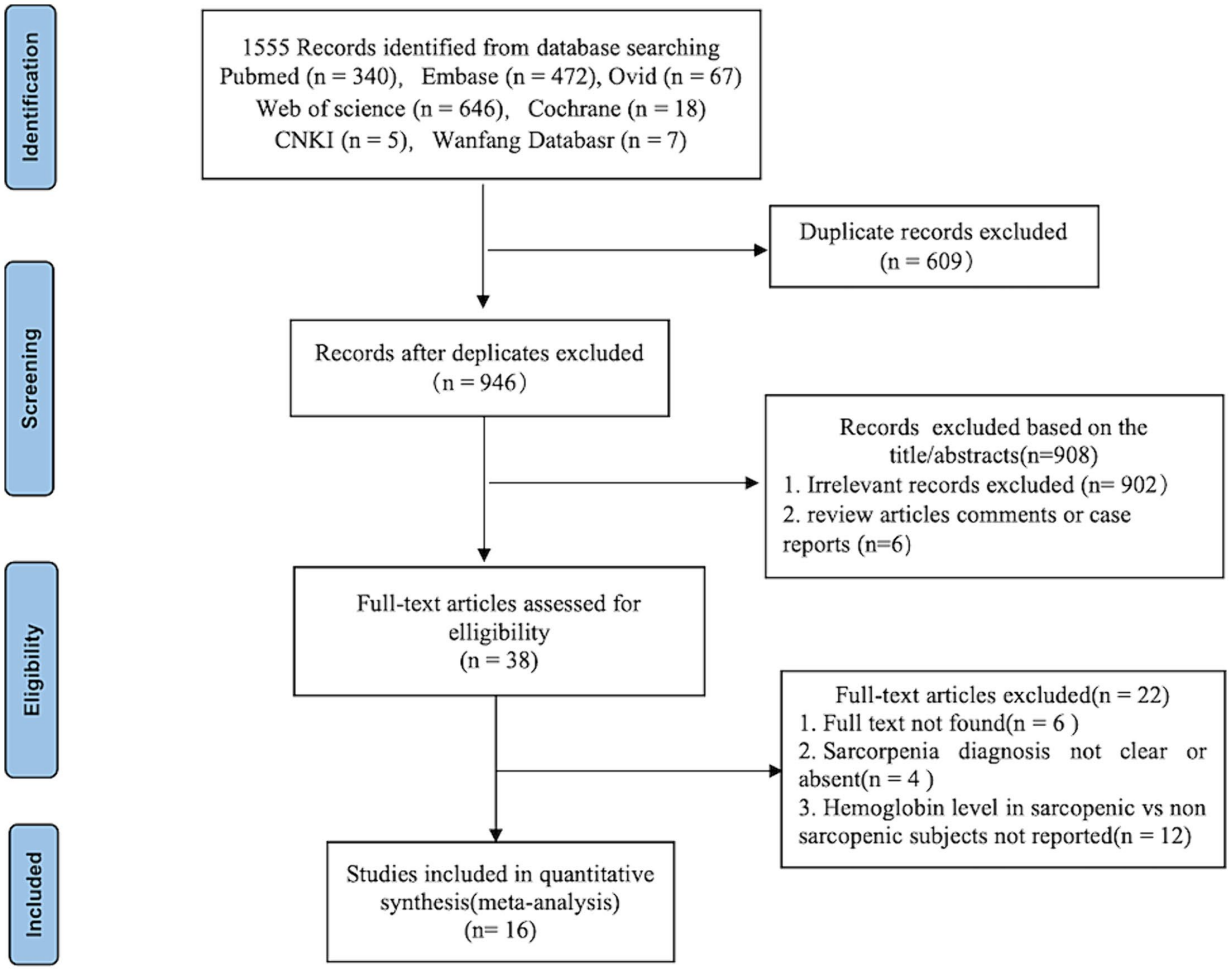
PRISMA flowchart of study selection.

After a comprehensive search, 1,555 published studies were identified from 7 databases. After removal of duplicates, 946 studies remained. After reading the title and abstracts, 38 studies were eligible for full-text review and data assessment. From these, 6 studies did not find full text, 12 studies did not report quantitative expression of hemoglobin level in sarcopenic and non-sarcopenic subjects, other 4 studies reported diagnostic criteria for sarcopenia were not clear or absent. Finally, we included 16 studies in the meta-analysis. Figure 1 shows the study flow-chart.

### 3.2 Study characteristics

Table 1 shows the study characteristics of the included studies. A total of 35,746 individuals with 18,187 male and 17,541 female, 10,836 sarcopenia and 24,910 control. Studies were conducted in various countries, including China, Istanbul, and the United States of America. Fifteen studies employed a cross-sectional study (16–28), and one study was case control study (29). Seven studies (12, 14, 16, 18, 21, 23, 26) included community-dwelling individuals, while nine studies included hospitalized patients (17, 19, 20, 22, 24, 25, 27–29). Ten of the selected studies used the Asian Working Group for Sarcopenia (AWGS) definition of sarcopenia (12, 14, 16, 18, 19, 23–27), two studies identified sarcopenia through European Working Group on Sarcopenia in Older People (EWGSOP) (17, 21), and four studies identified sarcopenia by skeletal muscle mass index (SMI) (20, 22, 28, 29).

**Table 1:** Main characteristics of studies included in the meta-analysis.

Study	Study region	Study design	Setting	Age	Sample size	Sex (male/female)	Definition of sarcopenia	Diagnostic criteria
Dai (2023)	China	Cross-sectional	Community dwelling	≥60	5,016	2,575/2,441	Low muscle mass coupled with low muscle strength or poor physical performance	AWGS
Gulcicek (2023)	Istanbul	Cross-sectional	Hospital	≥18	220	117/103	Low grip strength, low muscle mass	EWGSOP
Hai (2017)	China	Cross-sectional	Community dwelling	≥60	836	415/421	Low muscle mass with low muscle strength or low physical performance	AWGS
He (2020)	China	Cross-sectional	Hospital	≥50	1,125	586/539	Low muscle strength	AWGS
Hu (2024)	China	cross-sectional	Hospital	≥18	212	153/59	SMI-L3 <44.77 cm^2^/m^2^ in men and SMI-L3 <32.5 cm^2^/m^2^	SMI-L3
Kin (2018)	USA	Cross-sectional	Community dwelling	≥20	11,761	5,965/5,796	Lower muscle mass, lower walking speed	EWGSOP
Ko (2021)	China, Taiwan	Cross-sectional	Community dwelling	≥65	500	235/265	Low muscle mass, low grip strength, a slow walking speed	AWGS
Lee (2020)	Korea	Cross-sectional	Hospital	≥18	79	58/21	SMI-L3 <49 cm^2^/m^2^ in men and SMI-L3 <31 cm^2^/m^2^	SMI-L3
Liu (2023)	China	Cross-sectional	Community dwelling	≥60	3,055	1,572/1,483	Low muscle mass, low muscle strength, low physical performance	AWGS
Lu (2022)	China	Cross-sectional	Hospital	≥60	441	262/161	Loss of muscle mass plus low muscle strength and/or low physical performance	AWGS
Sun (2023)	China	Cross-sectional	Hospital	≥60	543	269/274	Low muscle mass and low muscle strength or low physical performance	AWGS
Tseng (2021)	China, Taiwan	Cross-sectional	Community Dwelling	≥50	730	386/344	Low muscle mass in combination with reduced muscle strength and/or low physical performance	AWGS
Wu (2021)	China	Cross-sectional	Community Dwelling	≥60	6,172	3,070/3,102	Low muscle mass plus low muscle strength or low physical performance	AWGS
Yao (2022)	China	Case control study	Hospital	18–80	259	179/80	Both decreased muscle mass and strength	AWGS
Zeng (2022)	China	Cross-sectional	Hospital	≥20	4,673	2,271/2,271	ASMI <7.23 kg/m^2^ in men and ASMI <5.67 kg/m^2^ in women	SMI
Zhang (2021)	China	Cross-sectional	Hospital	≥18	124	74/50	SMI <41 cm^2^/m^2^ in women; <43 cm^2^/m^2^ in men with BMI <25 kg/m^2^; <53 cm^2^/m^2^ in men with BMI ≥ 25 kg/m^2^	SMI-L3

AWGS, Asian Working Group for Sarcopenia; EWGSOP, European Working Group on Sarcopenia in Older People; SMI, skeletal muscle index; ASM, appendicular skeletal muscle mass; ASMI, appendicular skeletal muscle mass index.

### 3.3 Quality assessment of included studies

Table 2 shows the methodological quality of the included studies based on the NOS. The overall quality of the literature was considered strong, as the NOS score for case-control studies was 7, while the NOS scores for cross-sectional studies were all more than 4.

**Table 2:** Results of the Newcastle-Ottawa Scale quality assessment.

Case-control studies									
Author, year	Selection				Comparability (comparability of cases and controls on the basis of the design or analysis)	Exposure			Total
	Adequate definition of case	Representativeness of the cases	Selection of controls	Definition of controls		Ascertainment of exposure	Same method of ascertainment for cases and controls	Non-response rate	
Yao (2022)	1	1	1	1	1	1	1	0	7

Cross-sectional studies							
Author, year	Selection			Comparability (confounding factors are controlled)	Exposure		Total
	Representativeness of the sample	Selection of the non-exposed subjects	Ascertainment of exposure		Assessment of outcome	Response rate	
Dai (2023)	1	1	1	1	1	0	5
Gulcicek (2023)	1	1	1	1	1	0	5
Hai (2017)	1	1	1	2	1	0	6
He (2020)	1	1	1	2	1	0	6
Hu (2024)	1	1	1	1	1	0	5
Kin (2018)	1	1	1	1	1	0	5
Ko (2021)	1	1	1	1	1	0	5
Lee (2020)	1	1	1	2	1	0	6
Liu (2023)	1	1	1	2	1	0	6
Lu (2022)	1	1	1	1	1	0	5
Sun (2023)	1	1	1	1	1	0	5
Tseng (2021)	1	1	1	1	1	0	5
Wu (2021)	1	1	1	0	1	0	4
Zeng (2022)	1	1	1	1	1	0	5
Zhang (2021)	1	1	1	0	1	0	4

### 3.4 Hemoglobin expression and meta-analysis findings

**Figure 2:**
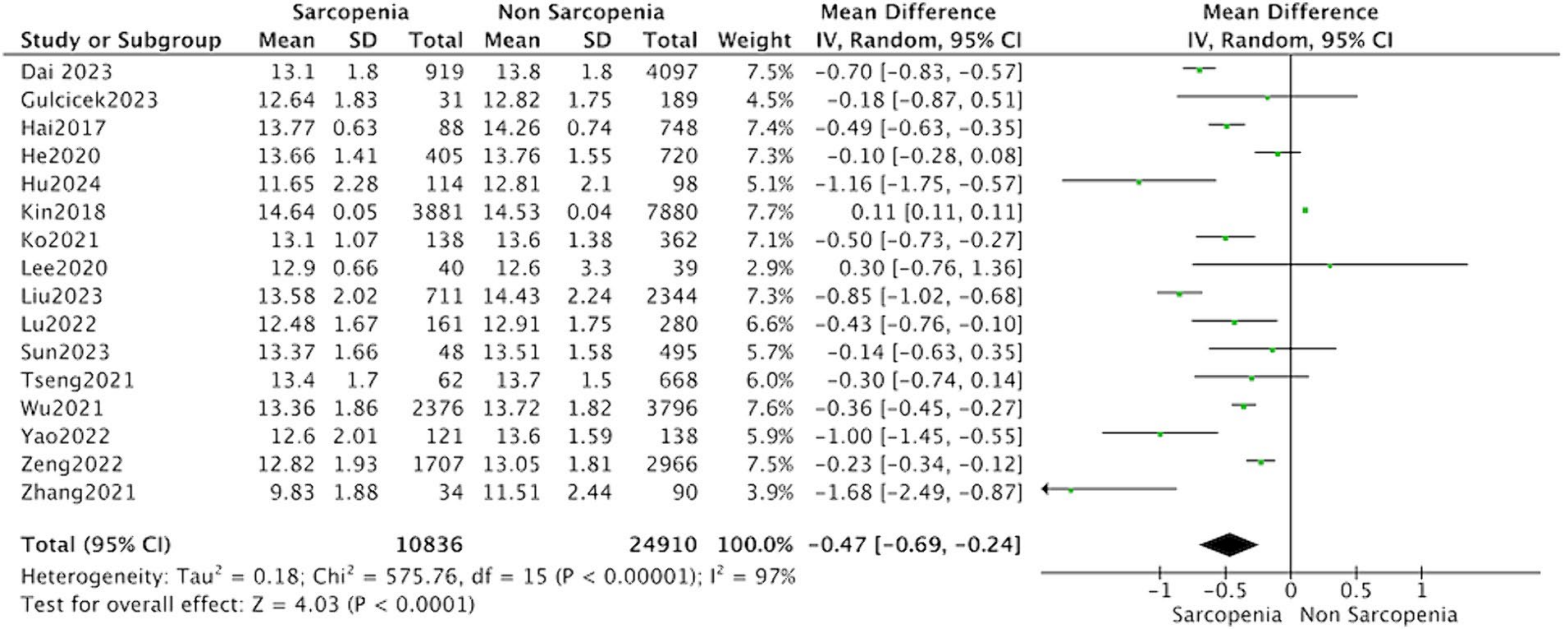
Forest plot of serum hemoglobin levels in sarcopenic vs. no sarcopenic subjects. SD, standard deviation; CI, confidence interval.

All 16 studies reported the levels of hemoglobin in patients with sarcopenia and in control subjects. The heterogeneity between the included studies was significant, so the random-effects model was applied. Meta-analysis of all included studies revealed that individuals with sarcopenia (*n* = 10,836), compared to individuals without sarcopenia (*n* = 24,910), were more likely to have significantly lower hemoglobin levels: (MD = −0.47, 95% CI: −0.69 to −0.24, *p* < 0.0001) (Figure 2). Meanwhile, subgroup analysis was performed to analyze the origin of the heterogeneity.

### 3.5 Subgroup analysis

**Figure 3:**
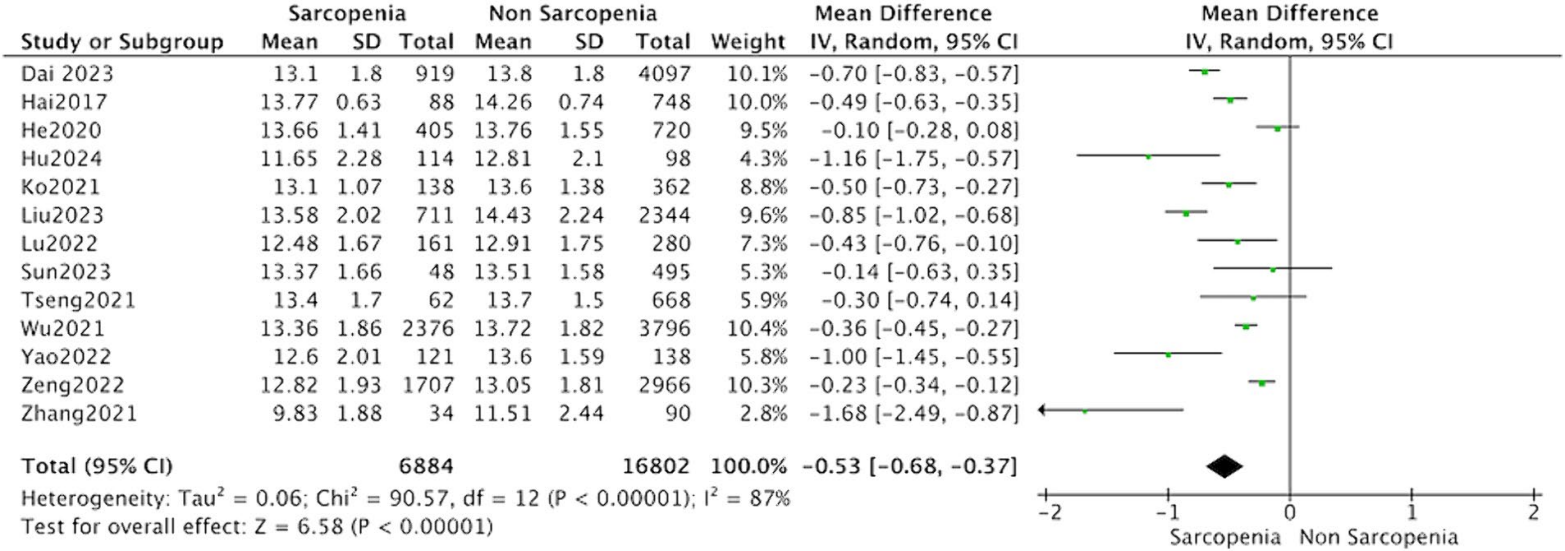
Forest plot of serum hemoglobin levels in sarcopenic vs. no sarcopenic subjects. Studies performed on China population. SD, standard deviation; CI, confidence interval.

**Figure 4:**
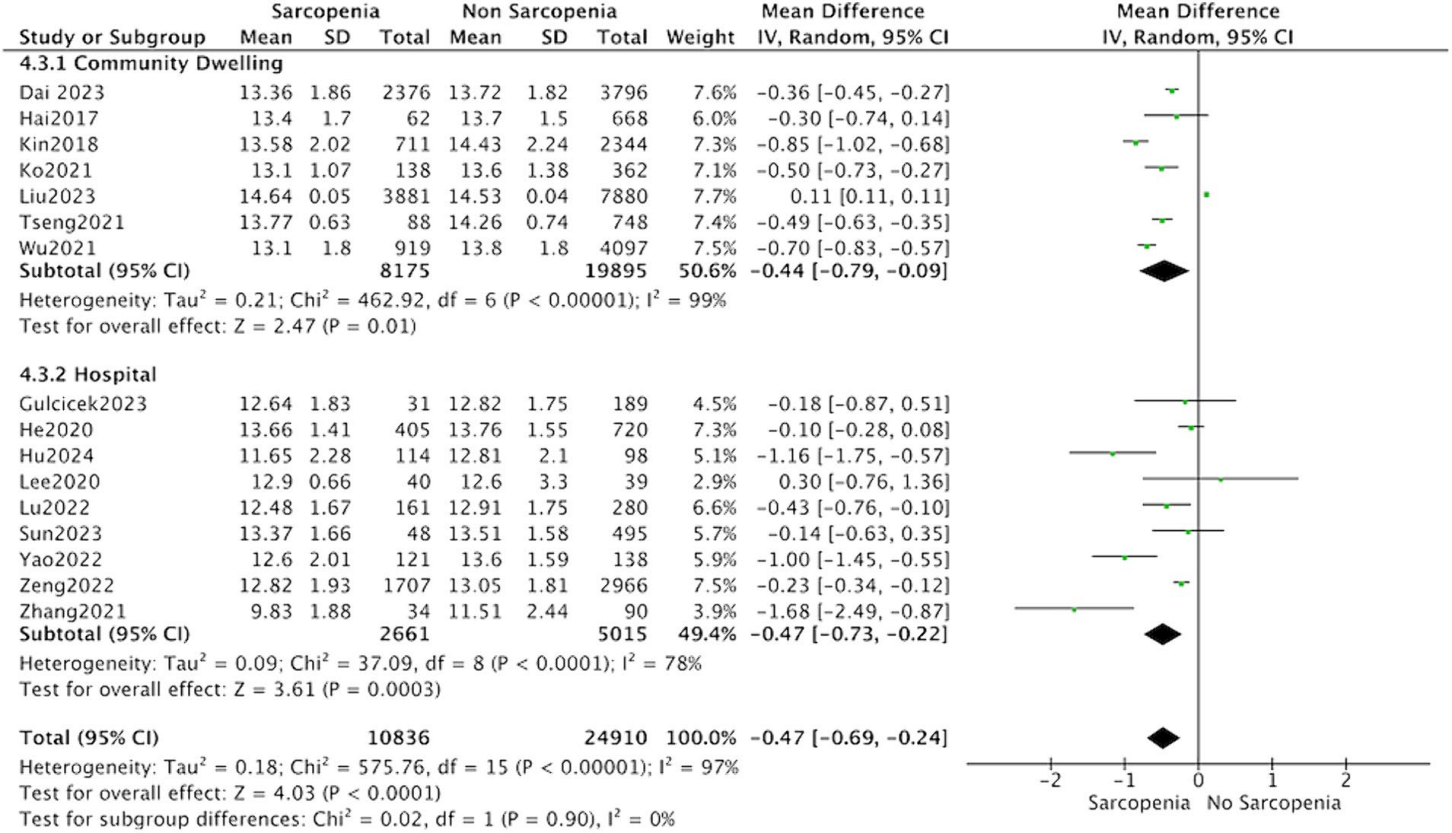
Forest plot of serum hemoglobin levels in sarcopenic vs. no sarcopenic subjects. Studies based on different study population. SD, standard deviation; CI, confidence interval.

Thirteen studies reported the hemoglobin levels in Chinese patients with sarcopenia and in control subjects. The heterogeneity between the included studies was significant, so the random-effects model was applied. The data suggested that Chinese patients with sarcopenia had lower hemoglobin levels (MD = −0.53, 95% CI: −0.68 to −0.37, *p* < 0.001) (Figure 3). Seven studies in community-dwelling and nine studies in hospitalized reported the hemoglobin levels patients with sarcopenia and in control subjects. The data suggested that among the population from hospitals and communities, patients with sarcopenia have lower hemoglobin levels (Figure 4).

**Figure 5:**
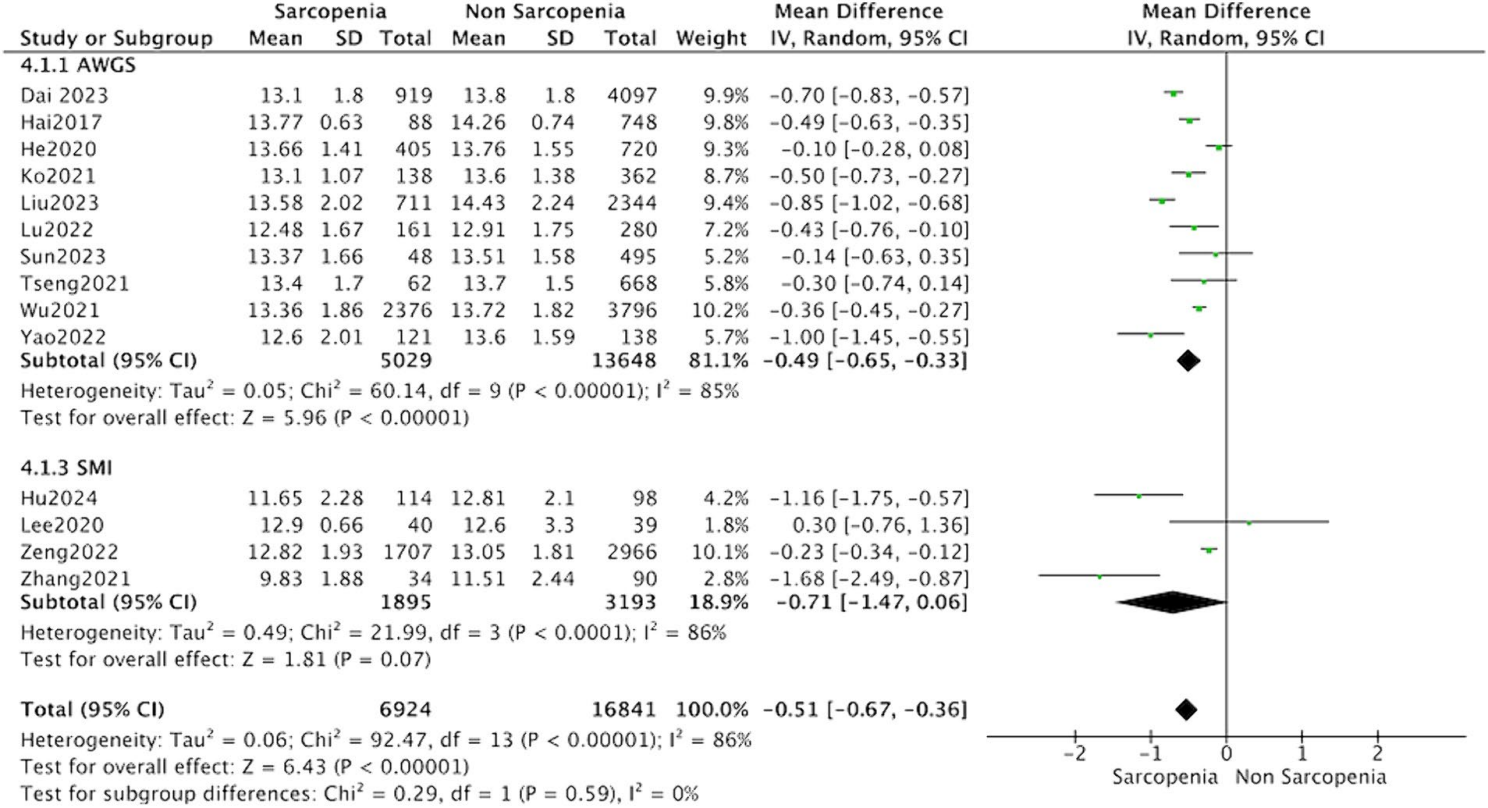
Forest plot of serum hemoglobin levels in sarcopenic vs. no sarcopenic subjects. Studies based on different diagnostic criteria for sarcopenia. SD, standard deviation; CI, confidence interval.

The subgroup analysis showed that the MD between hemoglobin and sarcopenia was −0.49 (95% CI: −0.65 to −0.33, *p* < 0.001) in the group diagnosed with sarcopenia according to AWGS guidelines. However, the subgroup analysis of studies based on AMI evaluation of sarcopenia did not report significantly different hemoglobin levels (MD = −0.47, 95% CI: −0.69 to −0.24, *p* = 0.07) (Figure 5).

### 3.6 Publication bias

Asymmetry was observed by visual inspection of funnel plots. However, Egger’s regression test (*p* = 0.259) indicated no statistically significant publication bias among the studies in this meta-analysis.

## 4 Discussion

In this meta-analysis, we incorporated 16 studies encompassing 35,746 individuals, comprising 10,836 with sarcopenia and 24,910 controls. Our analysis revealed that hemoglobin levels were lower in individuals with sarcopenia in contrast to those in non-sarcopenia patients.

We conducted a subgroup analysis involving 13 studies on Chinese individuals, revealing lower hemoglobin levels in Chinese patients diagnosed with sarcopenia. However, there is an insufficient number of studies conducted in the United States or Istanbul to allow for additional subgroup analysis. The meta-analysis incorporated studies from both community residents (7 studies) and hospitalized patients (9 studies). Subgroup analysis revealed that the characteristics of the study population did not influence the association between sarcopenia and hemoglobin levels.

In subgroup analysis, we found that individuals diagnosed with sarcopenia according to AWGS criteria exhibited a decline in hemoglobin levels. Conversely, no significant variances in hemoglobin levels were observed among those diagnosed with AMI in the subgroup analysis. This phenomenon may be ascribed to the comprehensive nature of AWGS definitions, which encompass not only diminished muscle mass but also reduced physical performance and/or grip strength. In a prior cross-sectional investigation on the Taiwanese population, it was found that low hemoglobin levels correlated solely with muscle strength and walking speed, rather than muscle mass (12). The human skeletal muscle expresses the erythropoietin receptor (30). The body’s sensitivity to erythropoietin stimulating agents is independently correlated with skeletal muscle mass (31). It is possible that inadequate skeletal muscle mass resulted in reduced hemoglobin production, potentially introducing reverse causality in cross-sectional studies. In addition, since only 2 studies employed the EWGSOP diagnostic criteria, a subgroup analysis was not performed.

Diminished hemoglobin levels are an independent risk factor for increased mortality and decreased quality of life in elderly individuals. Previous research has shown a significant correlation between hemoglobin levels and reduced muscle mass and muscle strength in patients undergoing kidney transplantation (32). This is consistent with the results of our meta-analysis that the hemoglobin levels of sarcopenia patients were significantly reduced compared to the control group.

The impact of low hemoglobin on sarcopenia may be multifaceted. As is well known, hemoglobin is mainly responsible for binding with oxygen in the human body and transporting oxygen to various tissues throughout the body. Diminished hemoglobin levels may reduce oxygen delivery to cells or tissues, leading to skeletal muscle hypoxia and impacting muscle strength and functionality (33). Anemia can also reflect the nutritional level of the human body. Low hemoglobin indicates insufficient nutrient intake, hindering protein synthesis, leading to a decrease in muscle mass and strength, and hastening sarcopenia progression (34). In addition, anemia patients are prone to fatigue, leading to reduced physical activity and muscle function (35). The decrease in iron content in anemia patients affects mitochondrial metabolism and myoglobin synthesis, consequently impairing muscle performance (36, 37).

Chronic inflammation plays an important role in both anemia and sarcopenia. Studies have shown that pro-inflammatory cytokines are associated with loss muscle mass and decreased physical function in elderly individuals (38–40). The sustained inflammation can activate the NF-kB and TNF-α signaling pathways, promoting the pro-inflammatory cytokine like TNF-α, interleukin-6 (IL-6) mediates catabolism, leading to disrupted muscle protein balance, promoted cell apoptosis, and imped muscle repair and regeneration (41, 42). In addition, animal studies have revealed a negative correlation between cytokine levels and the extent of muscle atrophy (43). Scientific investigations have found that inflammatory cytokines such as IL-1, IL-6, and TNF-α in the body upregulate ferritin transcription through different signaling pathways, disturbing iron metabolism. This disruption inhibits red blood cell production, ultimately leading to anemia (44).

This study still has certain limitations. Firstly, we observed heterogeneity in all cross-sectional studies. However, the Meta-analysis of Observational Studies in Epidemiology guidelines indicate that heterogeneity is expected when analyzing observational data. Secondly, the incorporated research, comprising cross-sectional and case-control studies, lacks the capacity to establish causal relationships. Finally, there are differences in the diagnostic criteria for sarcopenia included. Despite our efforts in conducting subgroup analyses, the number of studies included in the subgroup analysis is relatively small, which may have caused false-negative results. In the future, it is imperative to conduct more longitudinal studies of high quality to delve into the correlation and potential mechanisms linking sarcopenia and anemia.

Despite these limitations, the current research finds suggest that sarcopenia appears to be linked to low hemoglobin level. These findings emphasize the importance of addressing both sarcopenia and hemoglobin levels in the elderly population to optimize health outcomes. Therefore, our findings may have significant implications for preventing sarcopenia and promoting healthy aging.

## 5 Conclusion

Our study shows that sarcopenia patients had significantly lower levels of hemoglobin than healthy people. Based on our subgroup analyses, we found that sarcopenia population in China had lower hemoglobin levels. And the characteristics of the study population did not influence the association between sarcopenia and hemoglobin levels. Nevertheless, considering the limitations of the included studies, further large-scale prospective investigations are warranted to validate these findings and elucidate the underlying mechanisms. These findings underscore the importance of addressing both sarcopenia and hemoglobin levels in the elderly population to optimize health outcomes.

## Data availability statement

The original contributions presented in the study are included in the article/supplementary material, further inquiries can be directed to the corresponding author.

## Author contributions

HW: Data curation, Formal analysis, Methodology, Writing – original draft. PL: Conceptualization, Data curation, Funding acquisition, Investigation, Writing – review & editing.
